# Supplementary material for: Relationships between Serotonin Transporter Availability and the Global Efficiency of the Executive Control Brain Network
Source: Int J Mol Sci. 2024 May 24;25(11):5713. doi: 10.3390/ijms25115713 (PMC11171774; doi:10.3390/ijms25115713)
Supplement: Supplementary file 1 [file ijms-25-05713-s001.zip › ijms-2965100-supplementary.pdf]

Table S1: Summary of the region of interest Included in the networks. They were downloaded from previous study (Shirer et al., 2012).

		Anatomical Location of Functional ROIs	Brodmann Areas
Default Mode Network	Dorsal	Medial Prefrontal Cortex, Anterior Cingulate Cortex, Orbitofrontal Cortex	9, 10, 24, 32, 11
		Left Angular Gyrus	39
		Right Superior Frontal Gyrus	9
		Posterior Cingulate Cortex, Precuneus	23, 30
		Midcingulate Cortex	23
		Right Angular Gyrus	39
		Left and Right Thalamus	N/A
		Left Hippocampus	20, 36, 30
		Right Hippocampus	20, 36, 31
	Ventral	Left Retrosplenial Cortex, Posterior Cingulate Cortex	29, 30, 23
		Left Middle Frontal Gyrus	8, 6
		Left Parahippocampal Gyrus	37, 20
		Left Middle Occipital Gyrus	19, 39
		Right Retrosplenial Cortex, Posterior Cingulate Cortex	30, 23
		Precuneus	7, 5
		Right Superior Frontal Gyrus, Middle Frontal Gyrus	9, 8
		Right Parahippocampal Gyrus	37, 30
		Right Angular Gyrus, Middle Occipital Gyrus	39, 19
		Right Lobule IX	N/A
Executive Control Network	Left	Left Middle Frontal Gyrus, Superior Frontal Gyrus	8, 9
		Left Inferior Frontal Gyrus, Orbitofrontal Gyrus	45, 47, 10
		Left Superior Parietal Gyrus, Inferior Parietal Gyrus, Precuneus, Angular Gyrus	7, 40, 39
		Left Inferior Temporal Gyrus, Middle Temporal Gyrus	20, 37
		Right Crus I	N/A
		Left Thalamus	N/A
	Right	Right Middle Frontal Gyrus, Right Superior Frontal Gyrus	46, 8, 9

		Right Middle Frontal Gyrus	10, 46
		Right Inferior Parietal Gyrus, Supramarginal Gyrus, Angular Gyrus	7, 40, 39
		Right Superior Frontal Gyrus	8
		Left Crus I, Crus II, Lobule VI	N/A
		Right Caudate	N/A
Salience Network	Anterior	Left Middle Frontal Gyrus	9, 46
		Left Insula	48, 47
		Anterior Cingulate Cortex, Medial Prefrontal Cortex, Supplementary Motor Area	24, 32, 8, 6
		Right Middle Frontal Gyrus	46, 9
		Right Insula	48, 47
		Left Lobule VI, Crus I	N/A
		Right Lobule VI, Crus I	N/A
	Posterior	Left Middle Frontal Gyrus	46
		Left Supramarginal Gyrus, Inferior Parietal Gyrus	40
		Left Precuneus	5
		Right Midcingulate Cortex	23
		Right Superior Parietal Gyrus, Precuneus	7, 5
		Right Supramarginal Gyrus, Inferior Parietal Gyrus	2, 40
		Left Thalamus	N/A
		Lobule VI	N/A
		Left Posterior Insula, Putamen	48
		Right Thalamus	N/A
		Lobule VI	N/A
		Right Posterior Insula	48
